# Investigating Antiarthritic Potential of Nanostructured Clove Oil (*Syzygium aromaticum*) in FCA-Induced Arthritic Rats: Pharmaceutical Action and Delivery Strategies

**DOI:** 10.3390/molecules26237327

**Published:** 2021-12-02

**Authors:** Faiyaz Shakeel, Prawez Alam, Abuzer Ali, Mohammed H. Alqarni, Abdullah Alshetaili, Mohammed M. Ghoneim, Sultan Alshehri, Amena Ali

**Affiliations:** 1 Department of Pharmaceutics, College of Pharmacy, King Saud University, Riyadh 11451, Saudi Arabia; salshehri1@ksu.edu.sa; 2 Department of Pharmacognosy, College of Pharmacy, Prince Sattam Bin Abdulaziz University, Al-Kharj 11942, Saudi Arabia; prawez_pharma@yahoo.com (P.A.); m.alqarni@psau.edu.sa (M.H.A.); 3 Department of Pharmacognosy, College of Pharmacy, Taif University, Taif 21944, Saudi Arabia; abuali@tu.edu.sa; 4 Department of Pharmaceutics, College of Pharmacy, Prince Sattam Bin Abdulaziz University, Al-Kharj 11942, Saudi Arabia; a.alshetaili@psau.edu.sa; 5 Department of Pharmacy Practice, College of Pharmacy, AlMaarefa University, Ad Diriyah 13713, Saudi Arabia; mghoneim@mcst.edu.sa; 6 Department of Pharmaceutical Chemistry, College of Pharmacy, Taif University, Taif 21944, Saudi Arabia; amrathore@tu.edu.sa

**Keywords:** clove oil, nanocarrier, microfluidization, Freund’s complete adjuvant, rheumatoid arthritis

## Abstract

The combined application of clove oil in a lipid nanocarrier opens a promising avenue for bone and joints therapy. In this study, we successfully developed a tunable controlled-release lipid platform for the efficient delivery of clove oil (CO) for the treatment of rheumatoid arthritis (RA). The ultra-small nanostructured lipid carriers co-loaded with CO (CONCs) were developed through an aqueous titration method followed by microfluidization. The CONCs appeared to be spherical (particle size of 120 nm), stable (zeta potential of −27 mV), and entrapped efficiently (84.5%). In toluene:acetone:glacial acetic acid (90:9:1 percent *v*/*v*/*v*) solvent systems, high-performance thin layer chromatography (HPTLC) analysis revealed the primary components in CO as eugenol (*R*_F_ = 0.58). The CONCs greatly increased the therapeutic impact of CO in both in vitro and in vivo biological tests, which was further supported by excellent antiarthritic action. The CONC had an antiarthritic activity that was slightly higher than neat CO and slightly lower than standard, according to our data. The improved formulation inhibited serum lysosomal enzymes and proinflammatory cytokines while also improving hind leg function. This study provides a proof of concept to treat RA with a new strategy utilizing essential oils via nanodelivery.

## 1. Introduction

Arthritis, or joint inflammation, is a chronic condition caused by a disruption in the production of pro-inflammatory cytokines and enzymes [1]. Rheumatoid arthritis (RA) is an autoimmune illness that affects several joints bilaterally and is characterized by increasing tenosynovitis (inflammation of a tendon), which leads to cartilage degradation, bone erosion, and eventually disability [2,3]. In terms of males to females, the prevalence of RA has been found to be 1:3, with prevalence ranging from 0.4 percent to 1.3 percent of the population depending on both sexes worldwide [4].

Analgesics, nonsteroidal anti-inflammatory medicines (NSAIDs), immunosuppressive glucocorticoids (ISGs), anticytokines (ACs), and disease-modifying antirheumatic medications (DMARDs) are currently accessible pharmaceuticals [3]. However, exceptional progress has been observed in the long-term development of DMARDs, which include (a) traditional DMARDs such as methotrexate, hydrochloroquine, and sulfadiazine, (b) targeted synthetic DMARDs, such as tofacitinib (pan-JAK) inhibitor) and baricitinib (JAK1/2-inhibitors), and (c) biologic DMARDs, such as infliximab tumor necrosis factor-α (TNF-α) inhibitor), etanercept (TNF-receptor inhibitor), siltuximab (interleukin-6 (IL-6) inhibitor), tocilizumab (IL-6R inhibitor), rituximab (B-cell depleting antibody), and abatacept (co-stimulatory molecules inhibitor) specifically utilized for this purpose [5,6,7]. In summary, NSAIDs, ISGs, ACs, and DMARDs have been shown to have low success rates in the treatment of RA, as well as significant side effects and substantial financial expense [5,8]. Exploration for safe molecules with low or no adverse reaction is therefore a major concern to treat the severity of RA. Herbs are always preferred over synthetic drugs due to their myriad benefits and triumph of popular therapeutic diversity [9,10].

In this study, the well-known herbal medicine *Syzygium aromaticum* was chosen as the subject of investigation. Clove bud (*Syzygium aromaticum*), a member of the Myrtaceae family, is regarded as one of the most powerful natural plants, containing a large amount of clove essential oil (CO). CO is considered as “Generally Regarded as Safe (GRAS)” category by the USFDA and therefore it is safe to use in pharmaceuticals within prescribed limits [11]. Studies revealed that CO has great antibacterial, antioxidant, analgesic, and anesthetic properties [12,13], perhaps due to the presence of eugenol (EU), eugenol acetate, and β-caryophyllene as the major components advocating various therapeutic effects [12,14]. Eugenol (4-allyl-2-methoxyphenol) is a major phenolic compound that accounts for 40–95 percent of CO [15,16] and has anti-inflammatory properties [17,18], which could be due to its ability to block the nuclear factor-kappa B (NF-B) signaling pathway, which is responsible [19]. Therefore, we can clinically correlate EU as a herbal substitute of DMARDs for the purpose of targeting inflammation and progressive tenosynovitis. At the same time, eugenol can affect skin penetration as several investigations have suggested its specific role in transdermal drug delivery as a penetration enhancer [20]. These essential oil-based medications are said to have a low risk of major side effects, are inexpensive, and are easily available to consumers [9,10]. 

The high volatility and low aqueous solubility of CO restricts its clinical uses and further confines the pharmaceutical development program. However, the nanotechnological contrivances may favor an option to overcome these drawbacks by encapsulating these active ingredients into nanocarriers [21]. Myriad applications of nanoformulations have been seen in recent years for herbal drugs, extracts, and essential oils, which ultimately provided additional benefit and increased patient compliance [22,23]. Nanocarriers offer advantages including the ability to encapsulate hydrophobic essential oils, their controlled release, drug penetration across the skin, thermodynamic stability, improved bioavailability, easy scale-up, and low cost of manufacturing [24,25]. Because lipid nanocarriers are made from physiological and biodegradable lipids such as triglycerides and fatty acids, they are deemed harmless (in GRAS category) [26,27]. Because of the increased lengths between fatty acid chains, lipid molecules in nano-lipidic carriers (NLCs) generate ample space for drugs to lodge, and so enable them to better entrap hydrophobic essential oils, making them appropriate for skin delivery [28,29]. Additionally, NLCs containing bioactive chemicals have the added benefit of preventing medication degradation and improving drug penetration through the skin [24,27,29].

Oral self-nanoemulsifying drug delivery systems have increased the biological activities of different essential oils, including CO, eucalyptus oil, and *Piper cubeba* essential oil, such as wound healing properties [13,30,31]. The topical delivery and bioactivity of niaouli essential oil has also been increased using a nanoemulsion technique [32]. Recently, some innovative techniques based on the application of supercritical carbon dioxide for the controlled and targeted drug delivery systems have been investigated [33,34,35]. However, the antiarthritic potential of nanostructured CO has not been studied via a topical drug delivery system. Therefore, the purpose of the study was to develop a lipid nanocarrier of CO and to investigate its potential for arthritis by imposing the topical route. We adopted an aqueous titration method followed by high shear mixing for formulation development. A high-performance thin layer chromatography (HPTLC) method was employed for all analyses of the marker compound as it is a simple and economical method [36]. Pharmaceutical evaluation, product stability, and biological studies were performed as per previously published reports [2,32,37]. According to the findings, designing appropriate nanolipidic carriers could be a successful method to improve CO benefits in RA.

## 2. Results and Discussion

### 2.1. Analytical Method

**Figure 1:**
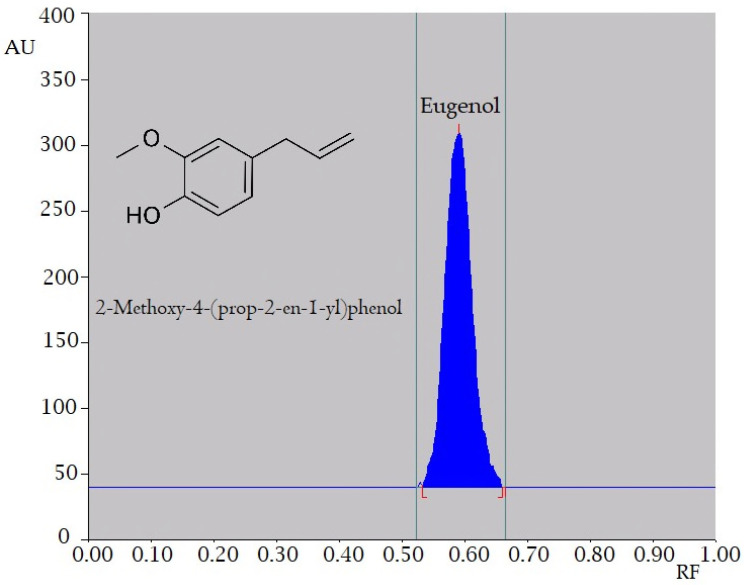
High-performance thin layer chromatography (HPTLC) chromatogram of eugenol (EU) (*R*_F_ = 0.58) in toluene:acetone:glacial acetic acid (90:9:1, *v*/*v*/*v*) solvent systems.

We adopted the high-performance thin-layer chromatography (HPTLC) method (CAMAG, Switzerland) as illustrated in the “materials and method” section for the quantification of EU in prepared formulations and as the crude oil (CO). A sharp chromatogram (*R*_F_: 0.58 ± 0.02) was observed by densitometric analysis in absorbance mode at 281 nm (Figure 1). The developed method was robust and accurate, therefore employed for all estimations.

### 2.2. Component Screening

CO was selected as an oil phase containing EU as a functional constituent for antiarthritic activity. Standard techniques were used to assess the solubility of CO in solid fats/triglycerides, surfactants, and cosurfactants [38]. CO was moderately miscible in lipids including stearic acid (SA), cetyl alcohol (CA), and glyceryl oleate (GO) at room temperature and below 10 °C. CO and GO in 1:1 ratio demonstrated better solubility performance (Table 1), therefore GO was selected as a solid lipid (base) for the study. The surfactant chosen for drug development must be able to (i) lower interfacial tension to a very low value to aid dispersion during nanoemulsion preparation, (ii) provide a flexible film that can easily deform around droplets, and (iii) have the appropriate lipophilic character to provide the correct curvature at the interfacial region for the desired nanoemulsion type (i.e., o/w, w/o, or bicontinuous) [32,38,39,40]. The surface area (mm^2^) as a function of phase behavior was determined for the CO in different surfactants and cosurfactants and the combination thereof to find out the best possibility towards formulation development. We reported the phasic surface area in mm^2^ for CO in Table 1, as Tween 80 (141.39 ± 2.13 mm^2^) > Tween 20 (119.56 ± 3.11 mm^2^) > Brij 20 (97.08 ± 4.26 mm^2^) > Cremophor-EL (77.10 ± 2.55 mm^2^) > ethanol (62.23 ± 2.06 mm^2^) > polyethylene glycol-400 (PEG-400) (53.12 ± 7.52 mm^2^) > PEG-200 (50.79 ± 4.26 mm^2^) > Labrasol (29.70 ± 2.55 mm^2^) > Labrafac (18.78 ± 3.44 mm^2^) > Transcutol-P (13.95 ± 2.82 mm^2^). Based on high solubility (phasic area) results, Tween 80 (surfactant; phase area ~141.39 ± 2.13 mm^2^) and Cremophor-EL (cosurfactant phase area ~77.10 ± 2.55 mm^2^) were selected for the lipid nanoemulsion preparation. 

### 2.3. Phase Diagram

**Figure 2:**
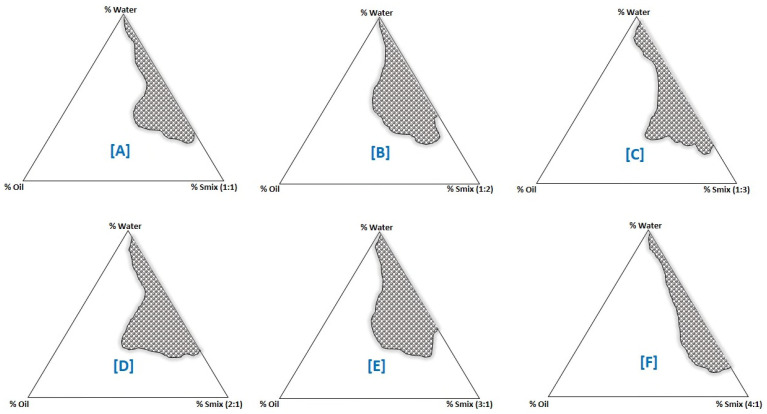
Phase diagrams demonstrating o/w nanoemulsion (shaded zone) region of oil (CO: GO; 1:1 *v*/*v*), surfactant (Tween-80), cosurfactant (Cremophor EL) at different S_mix_ ratios (**A**) (S_mix_ 1:1), (**B**) (S_mix_ 1:2), (**C**) (S_mix_ 1:3), (**D**) (S_mix_ 2:1), (**E**) (S_mix_ 3:1), and (**F**) (S_mix_ 4:1).

Constructing phase diagrams takes time, especially when the goal is to precisely define a phase boundary. In any case, the pseudo-ternary phase diagrams were created at a constant temperature using the aqueous titration method. Visual observation validated the production of mono/biphasic systems; in cases where turbidity developed, the formulation was regarded biphasic; nevertheless, in cases where the clean and transparent mixture was visible after stirring, the formulation was considered monophasic [40]. Phase diagrams were constructed separately for each surfactant to co-surfactant (S_mix_) combination (Figure 2) to identify o/w phases required for the optimization of lipid nanoemulsions. In addition to the water titration approach, the oil and water components were kept constant with varying surfactant/cosurfactant concentrations to complete the nanoemulsion domain. A large nanophasic area (o/w) appeared when a high concentration of S_mix_ was used. Tween 80 cannot be used alone, because it precipitates to undesired liquid crystals (LCs). For the above reason, cosurfactant was added to expand the nanoemulsion region and disrupt the LC formation. The phase diagram depicted the nanoemulsion domain obtained by these trials at various ratios of surfactant (Tween 80) to cosurfactant (Cremophor-EL) (Figure 2). 

The S_mix_ (1:0) was not able to break the interfacial tension with CO, confirmed by the presence of more LCs in the phase diagram. Adding Cremophor-EL (co-surfactant) to Tween 80 (surfactant) in 1:1 ratio (S_mix_), made the interfacial film more flexible to accommodate the drug and LCs started disappearing. The phase behavior investigation revealed that the greatest amount of oil that may be added to the nanoemulsion system was when the surfactant to cosurfactant ratio was 1:1. By adding S_mix_ approximately 60.7 ± 5.4 percent *v*/*v*, the largest amount of CO that solubilized was nearly 21.0 ± 1.2 percent *v*/*v*, according to the phase diagram (Figure 2A). It was discovered that as the cosurfactant concentration in the S_mix_ ratio (1:1, 1:2, and 1:3) increased, the LCs decreased, but the nanophases increased proportionately (Figure 2A–C). Inversely, raising the surfactant concentration in the S_mix_ ratio (2:1, 3:1, and 4:1) increased LCs, and phase diagrams revealed a tiny nanoemulsion area (Figure 2D–F). Because of the lower interfacial tension and increased fluidity at the interface, the system′s entropy increased somewhat, allowing more oil to be integrated into the hydrophobic area of surfactant monomers [40]. When the surfactant concentration in S_mix_ was increased from 2:1 to 3:1, the increase in the nanophase region was minimal (Figure 2D). Actually, this was the LC phase, attributed due to the presence of Tween 80 in high concentration which suppressed the effect of the cosurfactant. The above finding revealed that the free energy of nanoemulsion formation is more or less dependent on the extent to which the S_mix_ submissively reduces the interfacial tension of oil and water and dispersion entropy [38,40]. In such circumstances, the formation of nanoemulsions was spontaneous and produced physically stable dispersions [32,38,39,40]. Those formulations were selected from phase diagrams, which can accommodate a high quantity of oil with the low concentration of S_mix_.

### 2.4. Formulation Development

Different formulations from the nanoemulsion area were chosen from each phase diagram and screened further based on their performance during the thermodynamic stability test. The complete spectrum of nanoemulsion was covered by the phase diagrams, and the formula that used the lowest concentration of S_mix_ for nanoemulsion synthesis was chosen. The composition of the different lipid nanoemulsions is presented in Table 2. The oil (CO:GO; 1:1) was taken and a measured quantity of S_mix_ (Tween 80 and Cremophor EL) was added in the prescribed ratio. Following the slow titration method, the lukewarm water was then added dropwise till a clear and transparent solution was obtained. Microfluidization technology is a patented technology used to produce nanoparticles of desired size and features, therefore the obtained nanoemulsion was first solidified in cold water and then microfluidized (high shear homogenization) as illustrated earlier. 

### 2.5. Formulation Characterization

#### 2.5.1. Thermodynamic Stability

In vitro thermodynamic stability tests were undertaken to overcome the problem of the metastable form. The thermodynamic stability of the selected formulations, such as the centrifugation (C/T), heating and cooling cycles (H/C), and freeze-pump thaw (F/T) cycles, was tested. Table 2 shows the compositions of various formulations. The formulations that passed the thermodynamic stability tests were subjected to additional testing, including size, viscosity, and microscopy.

#### 2.5.2. Entrapment Efficiency (EE)

The EE stands for drug encapsulation capacity, which is an evident physicochemical property of lipid nanoparticles. Lipid nanocarriers (NLCs) have a better drug loading capacity and entrapment effectiveness than solid lipid nanoparticles (SLNs) [28,29]. CO was encapsulated in large amounts in the investigated CONC, which was attributed to superior lipophilic drug solubilization in the combination of oil and lipids (less ordered recrystallization) than in oil alone (Table 3). In the present study, the percentage entrapment of the best formulation was 91.34 ± 3.29. The combination of CO with GO led to high entrapment efficiency, i.e., 63.52% (CONC1); 72.30% (CONC2); 69.88% (CONC3); 82.51% (CONC4); 91.35% (CONC5), and 79.13% (CONC6), perhaps due to their lipophilic character and better loading capacity in stabilized formulations. 

#### 2.5.3. Electron Microscopy

**Figure 3:**
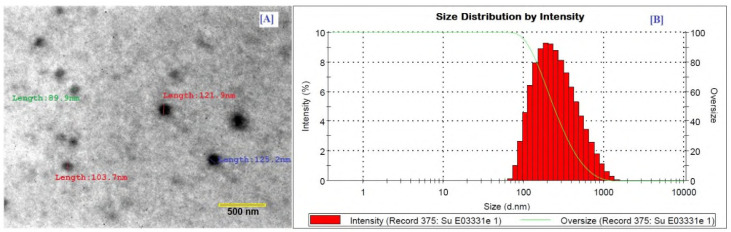
CONC5 formulation: (**A**) TEM image and (**B**) particle size intensity.

The morphological evaluation was performed by transmission electron microscopy (TEM) on a trial nanoformulation using “electron microscope JEM 1400-Plus (JEOL Ltd., Tokyo, Japan)”. CONC5 particles were found to be spherical in form using TEM imaging (Figure 3). The particles appeared dark with a bright surrounding in a positive image under TEM (Table 3). TEM imaging finally concluded the uniform and spherical CO nanoparticles with varying sizes, ranging approximately from 120–200 nm.

#### 2.5.4. Size and Charge

The Nano-ZS zetasizer was used to analyze the size distribution and surface charge of selected formulations (Malvern Instruments). The size and charge results of selected nanoformulations are represented in Table 3 and Figure 3. The largest droplet size appeared in CONC2, perhaps due to the presence of a high surfactant concentration which might lead to the formation of a rigid interface. An inadequate quantity of cosurfactant was found to be unable to provide further flexibility to the rigid film for secondary nanosizing. The mean particle size of the optimal formulations (CONC1–CONC6) ranged from 120 to 190 nm, with PDI values of less than 0.2, indicating that the system has a relatively narrow size distribution. It was observed that particle size increases with an increase in concentration of oil and S_mix_ ratio. CONC5 claimed the lowest PDI of 0.148, indicating a narrow distribution of size. A zeta potential of ±30 implies that nanodroplet/nanoparticle production is stable [40,41]. CONC6 formulation showed the highest ZP magnitude (−31.12 ± 2.4 mV), which fluctuated greatly, even after three months of storage at a regulated temperature. Finally, it was determined that the CONC with highly charged surfaces was stable and would resist droplet aggregation in a controlled temperature environment (45 °C/75%).

#### 2.5.5. Rheology

Table 3 shows the measured viscosity values of the optimized formulations, which ranged from 110 to 165 cps. Viscosity results were correlated with various surfactants and co-surfactant used in the stabilization of nanoformulation compositions. CONC5 was found to have a moderate viscosity of 142.73 cps as compared to others, suitable for topical administration.

### 2.6. Drug Release

**Figure 4:**
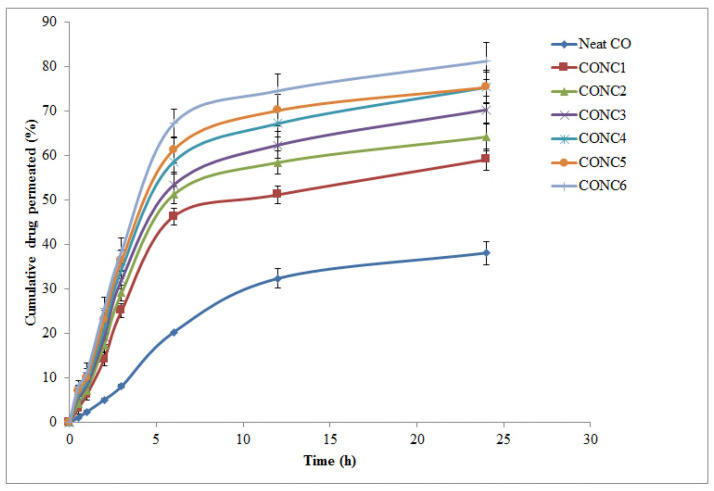
In vitro drug release pattern of EU via dialysis bag (mean ± SD, *n* = 3) from different CONC formulations and neat CO.

In vitro drug release was performed by using a dialysis membrane fitted over a modified Franz diffusion cell. The study was designed to compare the drug release of the optimized formulation and a nonformulated clove oil. The in vitro release demonstrated a prolonged release (Figure 4). Drug release from CONC5 was rapid (81.24% in 24 h), however, the formulations exhibited a comparatively prolonged release pattern [CONC5 (81%) > CONC6 (79%) > CONC4 (75%) > CONC3 (70%) > CONC2 (64%) > CONC1 (59%)] in 24 h. On the other hand, the nonformulated CO (neat) exhibited sluggish drug release (38% in 24 h) as compared to the optimized CONC. 

### 2.7. Skin Permeation and Retention

The ex vivo drug transport showed enhanced skin permeation for the optimized formulations as compared to the control (neat CO). The maximum transdermal flux value was found to be 82.41 ± 12.39 μg/cm^2^/h (CONC5) over the control formulation (28.17 ± 10.29 μg/cm^2^/h) with the enhancement ratio of 6.34 across the rat’s skin, possibly due to least droplet size and low viscosity (Table 4). In fact, the lipid nanoemulsions improve EU penetration via the SC lipid pathway, in which neutral lipids are organized as bilayers with their hydrophobic chains facing each other to produce a lipophilic bimolecular facet [32,40]. The CONCs directly penetrate the SC, where they destabilize the bilayer structure due to the involvement of the present surfactant molecule leading to enhanced drug permeability. However, the hydrophilic domain hydrated the SC which led to enhanced percutaneous drug uptake. In terms of pharmaceutical considerations, a high initial flux is always seen as advantageous since a sufficient quantity of bioactives is rapidly released from the lipid nanocarriers to produce a prompt topical effect. The drug’s steady state flux (J_ss_), permeability coefficient (K_p_), and enhancement ratio (E_r_) were dramatically raised via the rat skin, possibly due to the synergistic actions of EU and surfactant/cosurfactant combinations. 

### 2.8. Product Stability

The factual changes in the product stability evaluation have been presented in Table 5. The average particle size of formulation CONC5 was not much affected after three months of storage, however other formulations were aggregated/clumped (at a regulated temperature) and showed an increase in particle size. Due to the protective ability of Tweens in regulated temperature and humidity conditions, the particles remained stable in a normal contour. Surfactant ability is one of the solid reasons for product stability. In general, aggregation accelerated with an increase in storage temperatures due to the physical destabilization of CONC as a consequence of energy input by the successively higher temperatures. The energy input amplified the system’s kinetic energy which led to particle collision, and then, finally, the aggregation of particles. 

### 2.9. Pharmacological Evaluation

#### 2.9.1. In Vitro Antiarthritic Activity

The antiarthritic effects of the produced nanoformulations in vitro were investigated using the protein denaturation method [42]. According to the literature, autoantigen production (in the case of RA) may be caused by protein denaturation. As a result, protein denaturation is one of the most common signs of arthritic inflammation. For this purpose, CONCs were compared with standard (Voltaren; diclofenac gel) products and analyzed. The results revealed that the standard products showed a percentage inhibition of 86.91, however, CONC showed 82.73 percent inhibition. The above finding demonstrated the antiarthritic effect of our developed nanoformulations. 

#### 2.9.2. Freund’s Complete Adjuvant (FCA)-Induced Arthritis in Rats

The volume of the ipsilateral (injected) and contralateral (noninjected) paws increased gradually after subplantar FCA injection in the left hind paws of rats [43]. The rats′ paw volume was measured on the 0th, 7th, 14th, and 21st days after the study began. The paw volume of all groups increased from day 0 to day 7, according to the findings. The results of the CONC5 formulation were equivalent to those of the standard (Voltaren gel), indicating that the new formulation is therapeutically effective (*p* < 0.01). The toxic group, on the other hand, experienced a constant increase in paw volume as a result of being the nontreatment group. As shown in Table 6, both the standards and the CONC5 formulation resulted in a significant reduction in paw volume throughout the second–third week. The treatment with CONC5 demonstrated enhanced cartilage regenerative activity compared with untreated or CO-free NC treatment groups. In FCA-induced arthritic rats, the examined formulation was shown to limit the immune response more significantly (*p* < 0.01) than neat CO, possibly because of its capacity to reduce acute inflammation by decreasing the inflammatory mediator’s cascade and then reducing vascular permeability. 

#### 2.9.3. Biochemical Analysis

Table 6 displays the FCA induced biochemical changes in “aminotransferase (AST), alanine transaminase (ALT), and alkaline phosphatase (ALP)” of treated and nontreated rats. In inflammatory progressions, the enzymes AST, ALT, and ALP play important roles in the production of physiologically active chemical mediators, such as bradykinins. The improved therapeutic efficacy and penetration of the optimized CONC formulation may be responsible for the good recovery in the provided state. The level of AST, ALT, and ALP enzymes increased in all groups treated with FCA. The increased level of serum enzymes in arthritic rats was dramatically lowered after treatment with CONC5 and Voltaren gel. However, when FCA-induced arthritic rats were treated with CONC formulation, the increased enzyme levels were significantly reduced (*p* < 0.01), and the impact was superior to plain CO.

#### 2.9.4. Proinflammatory and Serum Markers

Because proinflammatory cytokines (TNF-α and IL-6) have been shown to play a significant role in the development of RA, the blood levels of TNF-α and IL-6 cytokines in treated arthritic rats were determined and reported in Table 6. TNF-α and IL-6 levels were found to be considerably higher in FCA-induced arthritic rats (*p* < 0.01), whereas treatment with the CONC formulation effectively reduced these elevated levels. TNF-α (pleiotropic cytokine) is a pleiotropic cytokine produced by activated monocytes and macrophages that regulates immune cells and plays a key role in inflammation (both chronic and acute). TNF-α stimulates inflammatory responses, which can lead to autoimmune diseases such as ankylosing spondylitis and RA. IL-6, on the other hand, is said to harm synovial cells by increasing prostaglandin synthesis and fibroblast proliferation in the synovial fluid. As a result, we looked at the levels of TNF-α and IL-6 cytokines in the serum of arthritic rats. The levels of TNF-α and IL-6 in the FCA-induced arthritic rat were significantly raised, while treatment with CONC5 decreased the elevated levels of serum TNF-α and IL-6. The developed CONC formulation was found promising due to its potential to inhibit proinflammatory mediators by arresting TNF-α and IL-6 activation. The C-reactive protein (CRP) and rheumatoid factor (RF) levels in the blood are indicators of systemic inflammation and antibody formation against the adjuvant administered. The FCA control group rats had high levels of serum CRP (8.2 mg/L) and serum RF (57.9 IU/L). Both CRP and RF levels in the serum were dramatically lowered by the CONC5 and Voltaren gel treatments. Because the levels of TNF-α and IL-6 were significantly reduced by the topical administration of CONC5, it is expected that the levels of other proinflammatory biomarkers such as IL-4, IL-10, and IL-11 would also be reduced by the same formulation. Overall, this research suggested that CO in a CONC formulation significantly decreased the elevated levels of proinflammatory biomarkers, such as TNF-α and IL-6 in rats, leading to an anti-inflammatory effect. As a result, the anti-inflammatory potential of CO in a CONC formulation might be due to the inhibition of TNF-α and IL-6 by the topical administration in rats [44,45,46].

## 3. Materials and Methods

### 3.1. Drugs and Chemicals

The working standard of EU (purity: 99.8%), GO, Brij 98, isopropanol (IPA), and complete FCA were obtained from “Sigma Aldrich (St. Louis, MO, USA)”. CO was purchased from “Loba Chemie Pvt. Ltd., (Mumbai, India)”. Capryol-90, Transcutol-P, Labrasol, and Labrafac were obtained from “Gattefosse (Lyon, France)”. Cremophore-EL, CA, and SA were obtained from BASF (Darmstadt, Germany). Tween 80, Tween 20, PEG-200, and PEG-400 were obtained from “E-Merck (Darmstadt, Germany)”. HPLC grade methanol and ethyl alcohol were purchased from “Fluka Chemica (Darmstadt, Germany)”. Purified water was obtained from “Milli-Q water purification system (Millipore, Billerica, MA, USA)”. All additional chemicals and reagents used in the experiment were analytical grade and purchased from a reputable vendor.

### 3.2. Analytical Method

HPTLC method was employed for all analyses of marker compound as it is a simple and economical method [36]. The HPTLC approach was used to quantify an active ingredient of CO, namely EU (CAMAG, Muttenz, Switzerland). With a CAMAG microliter (µL) syringe, the EU samples were smeared in the shape of bands with a width of 5 mm on a precoated silica-gel aluminum plate 60F254 (20 cm × 10 cm with 0.2 mm thickness) (E-Merck, Darmstadt, Germany) using the CAMAG Linomat V sample applicator (CAMAG, Muttenz, Switzerland). The mobile phase was toluene:acetone:glacial acetic acid (90:9:1 *v*/*v*/*v*), and formed plates were dried and sprayed with vanillin-sulfuric acid reagent to visualize the zones. The CAMAG TLC scanner III was used to perform densitometric scanning in the absorbance mode at 281 nm. This method was employed for all analytical estimations pertaining to the study.

### 3.3. Phase Diagram

Pseudo-ternary phase titration was performed for CO at a constant temperature with one of the immiscible components to a turbidimetric end-point by employing conventional water titration method [32]. Concisely, the CO (oil) and GO (fat) were mixed in 1:1 mass ratio (melted on hot water bath) and combination of Tween 20 and Cremophor-EL (surfactant mixture) was dissolved in IPA (co-surfactant) by gentle stirring (Table 1). Surfactant/co-surfactant mixtures (S_mix_) were obtained in different volume ratios (1:0, 1:1, 1:2, 1:3, 2:1, 3:1, and 4:1) for the titration. Thereafter, various combinations of oily phase (CO:GO; 1:1) and specific S_mix_ were prepared in 9 volume ratios (1:9, 2:8, 3:7, 4:6, 5:5, 6:4, 7:2, and 9:1) to cover the maximum volume ratios, which allowed the boundary between the phases formed during the pseudo-ternary phase construction to be precisely delineated. Lukewarm purified water was used as the aqueous phase in the titration. A slow titration was performed for each combination (Oil:S_mix_) using lukewarm water as an external phase [38,39]. Subsequently, each set was visually examined for transparency and isotropy, as well as for boundaries between homogeneous and heterogeneous mixtures (i.e., single-phase, clear, fluid, and homogeneous nanoformulation). Pseudo-ternary diagrams with an aqueous phase on one axis, an oil phase on the other (CO:GO), and a specific mixture of surfactant/cosurfactant in set volume ratios on the third axis were created. The batches showing significant clarity and transparency were then transferred to cold aqueous medium (1–3 °C) and then homogenized with a microfluidizer (HG-15D DAIHAN Scientific Co., Ltd., Chuncheon, Korea) operating at 12,000 rpm for 15 min ensuring proper mixing of the system [47].

### 3.4. Formulation Development

We adopted the nanoemulsion technique, described by Muller et al., to prepare the CO nanocarriers (CONCs) [41], followed by microfluidization treatment [47]. In brief, from each constructed phase diagram, different formulations were chosen from the nanoemulsion region and selected for the study. Furthermore, when distributed in a cold aqueous media (2–3 °C) under light mechanical mixing, these lipid nanoemulsions recrystallized, ensuring that the small size of the particles formed was due to precipitation and not mechanically generated by the stirring process [41,48]. Therefore, microfluidization technique was employed to reduce the particle size of prepared formulation to as small as possible [49]. Finally, the produced lipid nanoemulsion (o/w) was stirred for 15 min on a high shear homogenizer at 12,000 rpm, and the resulting formulation was allowed to cool to room temperature.

### 3.5. Formulation Characterization

The developed nanoproduct was evaluated for various pharmaceutical properties as per the standard protocol mentioned below [37,38].

#### 3.5.1. Thermodynamic Stability

To observe the physical changes, a C/T was used to test the thermodynamic stability of sample formulations at 6000 rpm for 30 min (phase separation, creaming or cracking) [38]. Formulations passing the above test were subjected to six consecutive cycles of heating (45 °C) and cooling (0 °C) (H/C) 8 hourly for the next two days. Further, the H/C passed formulations were given six cyclic treatments of F/T in between the temperature range of −21 °C to 25 °C for 48 h. Out of the test samples, those which survived and passed all the above tests (C/F, H/C, and F/T) were finally selected for biological screening (Table 2).

#### 3.5.2. Entrapment Efficiency

The EE of the developed CONC was determined in terms of EU content. The sample was centrifuged (Thomas scientific centrifuge, 5418-R, Swedesboro, NJ, USA) at 12,000 rpm for 30 min to remove noncapsulated CO, and the supernatant was gently harvested and diluted with phosphate buffer (pH 7.4). The EU content was then calculated using the HPTLC method, as shown in the “analytical procedure section”. The percentage entrapment efficiency of the EU was determined by using the following formula:

(1) $$\%\mathrm{EE}\;=\frac{C_{t}-C_{r}}{C_{t}}\times 100$$

where, ‘C_t_’ is the total concentration of incorporated and nonincorporated EU in CONC and ‘C_r_’ is the concentration of free EU in CONC.

#### 3.5.3. Electron Microscopy

The size and surface morphology of the trial formulation were confirmed using a transmission electron microscope JEM 1400-Plus (JEOL Ltd., Tokyo, Japan) operating at a 200 kV acceleration voltage. The test formulation was diluted in water (1:100) and filtered through a membrane syringe filter with a cut off diameter of 0.2 µm for the TEM examination. After complete drying, a drop of the diluted CONC was gently placed on a circular copper grid (TAAB Laboratories Equipment, Berks, UK) and viewed under a microscope.

#### 3.5.4. Size and Charge

Dynamic light scattering (DLS) was used to determine particle size and size distribution using a Nano-ZS zetasizer (ZEN 3600, Malvern Instruments, Darmstadt, Germany) based on the laser light scattering phenomenon, which analyzes variations in light scattering. Prior to the size analysis, test samples were adequately diluted at 1:5 (CONC/water) and held at room temperature (25 °C) for 5 min. Zeta potential (ζ) was determined by Nano-ZS using the laser doppler velocimetry technique. Before being lodged in the instrument for charge measurements, test samples were diluted with KCl 1 mM (pH 7.0). Each measurement was carried out three times and the results were recorded individually as mean ± standard deviation (SD).

#### 3.5.5. Rheology

Viscidness of lipid nanoemulsion was measured using Brookfield Viscometer (DVELV Ultra, Brookfield Engineering Laboratories, Inc., Middleboro, MA, USA) at a constant room temperature (25 ± 0.3 °C) at 100 rpm. The calculations were interpreted by using Rheocalc software (Version #2.6). Effect of microfluidization (high shear homogenization) cycles on change of the viscosity value was determined in centipoises (cps).

### 3.6. Drug Release

CONC formulations were tested for in vitro drug release using a dialysis membrane method with a modified Franz diffusion cell (A: 7.16 cm^2^; V: 37 mL of receiver chamber) [50]. Dialysis membrane (2.4 nm, 12,000–14,000 Da) previously soaked with dissolution media (7:3; acetate buffer pH 5.4: ethanol) was mounted over Franz diffusion cell 12 h before the sample run. The release profile in dissolution media (pH 5.6) was also used to simulate cadaver skin pH. To run the experiment, CONC formulations were precisely weighed and placed in a donor compartment with receptor compartment filled with dissolution media. During the experiments, the solution on receptor side was kept at 37 °C ± 0.5 °C with stirring magnetic bead speed of 100 rpm. Aliquots (100 μL) were withdrawn from receiver compartment through side tube at a regular interval of time (0, 0.5, 1, 1.5, 2, 4, 6, 8, 10, 12, 24, 36, and 48 h) and substituted by an equal volume of fresh media each time. The samples were analyzed for the drug content using HPTLC method as illustrated earlier.

### 3.7. Skin Permeation and Retention

The Franz diffusion cell with a diffusion surface area of 7.16 cm^2^ and a receiver compartment capacity of 37 mL was used to analyze skin permeation on excised Wistar-rat abdomen skin. The full thickness skin was cleaned with purified water and stored in a deep freezer at −20 °C until it was used in the experiment. The skin was warmed to room temperature before being installed atop a diffusion cell in the middle of the donor and receiver compartments, with the stratum corneum (SC) side facing the donor compartment and the dermal side facing the receiver compartment. At 37 ± 1 °C, the receiver chamber was filled with ethanolic acetate buffer pH 5.4 (7:3) and stirred at 100 rpm. In the donor compartment, one mL of CONC formulation was inserted. At regular intervals (0.5, 1, 2, 3, 4, 5, 6, 7, 8, 9, 10, 12, 14, 16, 24, and 48 h), aliquots (1 mL) were taken from the receiver cell, filtered through a 0.45 µm membrane filter, and analyzed using HPTLC at 281 nm. For each formulation, the cumulative amount of medication penetrated through the skin (g/cm^2^) was plotted as a function of time (t). By dividing the slope of the linear component of the graph with the area of the diffusion cell, the J_ss_ was computed. Using the equation below, the K_p_ was computed by dividing J_ss_ by the initial concentration of drug in the donor cell (C_0_) [32]:

(2) $$K_{p}=\frac{J_{\mathrm{ss}}}{C_{0}}$$

Using the following equation, the E_r_ was computed by dividing the J_ss_ of the test formulation by the J_ss_ of the control formulation [32]:

(3) $$E_{r}=\frac{J_{\mathrm{ss}}\mathrm{of}\mathrm{CONC}\mathrm{formulation}}{J_{\mathrm{ss}}\mathrm{of}\mathrm{control}}$$

However, the skin retention study was performed to analyze the content of CO in the skin after 48 h of diffusion. At the end of experiment, the skin sample was washed with purified water and methanol on both sides respectively and then it was carefully dried. After that methanol (10 mL) was added to the skin and after vortexing for 10 min, the content was kept stirred overnight. The next day, the extracted sample was centrifuged and supernatant was analyzed by HPTLC at 281 nm. 

### 3.8. Product Stability

The product stability of developed CONC was examined to ensure the physicochemical stabilities, such as aggregation, precipitation, fusion, and degradation upon storage. In a nutshell, the CONC formulations were tested for particle size, zeta potential, and other characteristics over the course of three months (latest time-point evaluated) at 25 °C and 40 °C.

### 3.9. Pharmacological Evaluation

#### 3.9.1. Experimental Animals

Male Albino Wistar rats (180–220 g) of both sexes were used for the pharmacological evaluations of CONCs. The animals were obtained from “Experimental Animal Care Center (EACC) at Prince Sattam bin Abdulaziz University, Al-Kharj, Saudi Arabia”. The rats were housed under standard animal housing facilities (T = 24 ± 1 °C, %RH = 45–50%, and 12 h light/dark cycle), on standard pellet chow diet and water *ad libitum*. All experimental protocols and procedures were carried out in compliance with the guidelines of the “EACC, Prince Sattam bin Abdulaziz University, Al-Kharj, Saudi Arabia”. These studies were approved by the “Animal Ethics Committee of the EACC Board (Prince Sattam bin Abdulaziz University, Al-Kharj, Saudi Arabia with approval number: BERC-016-10-21)”. The use of animals and experimental protocols were in accordance with the “European Union (EU) directive 2010/63/EU”.

#### 3.9.2. In Vitro Antiarthritic Activity

In vitro antiarthritic potential of prepared CONCs were compared with topical Voltaren-diclofenac sodium gel (standard) by using protein denaturation method [42]. The reaction mixture (0.5 mL) was made up of 0.45 mL bovine serum albumin (5 percent aqueous BSA) and 0.5 g each of the test and control formulations, with the pH adjusted to 6.3 with 1N HCl. Both samples were incubated at 37 ± 1 °C for 15 min before being gently heated at 57 °C for 3 min. Following cooling, a 2.5 mL aliquot of PBS (pH 6.3) was added to each sample. The spectrophotometer was used to carefully measure turbidity at 660 nm, and the percentage inhibition (PI) of protein denaturation was computed using the formula below:

(4) $$\mathrm{PI}=\frac{\mathrm{OD}\mathrm{of}\mathrm{test}-\mathrm{OD}\mathrm{of}\mathrm{control}}{\mathrm{OD}\mathrm{of}\mathrm{control}}\times 100$$

#### 3.9.3. FCA-Induced Arthritis in Rats

The antiarthritic evaluation in rat model was performed for CONC formulation (test) and CO-free NC formulation (control) in accordance with our previous research on arthritis [2]. The rats were chosen at random and placed into four experimental groups (each including six rats) for the evaluation of FCA-induced arthritis:

Group I—CO-free NC was used as a topically applied control;
Group II—no pharmacological therapy was given to the toxic control group, which had been treated with FCA.;
Group III—treated group, which received 1.2 percent topically administered CO loaded NC (i.e., CONC);
Group IV—positive control was 1.16 percent *w*/*v* diclofenac gel applied topically, as per normal marketed formulation.

The research was scheduled to last three weeks. In the 1st week, all animals in all groups, except Group I—normal control, received 0.1 mL of FCA subcutaneously into the subplantar area of the left hind paw [43]. FCA is made up of *Mycobacterium tuberculosis* that has been heat destroyed, mineral oil, and mannide monooleate. The FCA suspension once injected may cause inflammatory reactions within 24 h. On days 1, 4, 7, 10, 12, 14, 17, and 21, the antiarthritic activities of test samples and standard were determined using a “Plethysmometer (Ugo, Basile, Italy)” by the hind paw method [43]. The volume of the left paw was measured using the mercury displacement method up to the lateral malleolus shortly before FCA injection on the first day, and thereafter using the plethysmometer at various time intervals until the 21st day. The difference between the end and starting paw volumes was used to calculate the changes in paw volume [2,43].

#### 3.9.4. Biochemical Analysis

Blood samples were taken from all groups of rats on the 21st day via retro-orbital puncture. Harvested blood samples were immediately transferred to the anticoagulation tubes and the required reagents were added precisely. A standard kit (Sigma-Aldrich assay kit) was used to measure various biochemical parameters such as AST, ALT, and ALP.

#### 3.9.5. Proinflammatory and Serum Markers

The blood samples were maintained undisturbed for around half an hour to estimate proinflammatory biomarkers such as TNF-α and IL-6. Centrifugation (3000 rpm, 10 min) was used to separate the serum from the blood, which was then stored at −20 °C until further analysis. TNF-α and IL-6 levels were measured using ready-to-use ELISA reagent kits (Biosource Inc., Camarillo, CA, USA) in accordance with established procedures. Routine laboratory techniques, on the other hand, were utilized to estimate serum parameters. Commercial kits (Aspen labs, England, UK) were used to quantify blood CRP and RF levels according to the manufacturer′s instructions.

### 3.10. Statistical Evaluation

Statistical analysis was carried out using one-way analysis of variance (ANOVA) with Tukey′s multiple comparisons test, with a statistical significance level of *p* < 0.05 (95 percent confidence interval) unless otherwise stated. Graph-pad prism software-VI was used to conduct all statistical analyses.

## 4. Conclusions

The CO-loaded NLCs were effectively synthesized using an aqueous titration approach followed by microfluidization, and their antiarthritic potential was studied in vitro and in vivo. The CONC was discovered to have a higher transdermal flux value across rat skin in the current investigation. The antiarthritic effects of CONC were comparable to those of the standard product, according to in vivo studies. With a fall in the biochemical parameters, the rat paw volume revealed a depleting effect. The findings demonstrated that the optimized formulation has inhibitory effects on enzymes and lowers IL-6 and TNF-α levels. The foregoing findings demonstrate that our CONC formulation was helpful in the treatment of arthritis.

## Figures and Tables

**Table 1:** Comparative phasic area of clove oil (CO) in various surfactants and cosurfactants (mean ± SD, *n* = 3).

Component	Phasic Area ± SD (mm^2^)
Tween 20	119.56 ± 3.11
Tween 80	141.39 ± 2.13
Brij 20	97.08 ± 4.26
PEG-200	50.79 ± 4.26
Ethanol	62.23 ± 2.06
PEG-400	53.12 ± 7. 52
Cremophor EL	77.10 ± 2.55
Labrasol	29.70 ± 2.55
Labrafac	18.78 ± 3.44
Transcutol-P	13.95 ± 2.82

**Table 2:** Composition of selected CONC that passed the physical stability tests.

Code	Selected Formulation (% *v*/*v*)			S_mix_ Ratio
	Oil (eq-CO:GO)	S_mix_	Water	
CONC1	10	40	50	1:1
CONC2	12	42	46	1:1
CONC3	10	39	51	1:2
CONC4	12	42	46	1:2
CONC5	10	39	51	1:3
CONC6	12	42	46	1:3

**Table 3:** CONC formulation physicochemical characterization (mean ± SD, *n* = 3).

Code	Diameter ± SD (nm)	PDI ± SD	Zeta Potential (mV) ± SD	Viscosity ± SD (cps)	EE ± SD (%)	Drug Release ± SD (%)
CONC1	131 ± 6.7	0.119 ± 0.017	−28.63 ± 1.7	157.87 ± 4.22	59.41 ± 7.66	46.14 ± 3.31
CONC2	187 ± 9.1	0.153 ± 0.022	−30.08 ± 2.1	163.27 ± 7.45	64.58 ± 6.72	58.62 ± 5.29
CONC3	163 ± 4.3	0.170 ± 0.051	−29.44 ± 2.8	138.31 ± 8.17	69.70 ± 9.35	64.08 ± 7.91
CONC4	144 ± 3.9	0.162 ± 0.034	−28.19 ± 3.6	135.92 ± 6.47	75.16 ± 7.61	72.38 ± 8.14
CONC5	120 ± 5.2	0.148 ± 0.013	−27.56 ± 1.9	142.73 ± 4.23	84.53 ± 4.12	81.24 ± 4.65
CONC6	156 ± 4.5	0.157 ± 0.044	−31.12 ± 2.4	157.36 ± 9.11	79.14 ± 3.56	57.26 ± 4.31

**Table 4:** In vitro permeation data of different CONCs (mean ± SD, *n* = 3).

Code	J_ss_ ± SD (mg/cm^2^/h)	K_p_ ± SD (cm/h × 10^−2^)	E_r_	*p*-Value *
Neat CO	28.17 ± 10.29	0.183 ± 0.15	-	-
CONC1	46.58 ± 09.66	0.513 ± 0.24	2.80	<0.05
CONC2	59.92 ± 11.34	0.867 ± 0.30	4.73	<0.05
CONC3	63.76 ± 12.49	0.909 ± 0.18	4.96	<0.01
CONC4	68.02 ± 10.51	1.215 ± 0.11	6.63	>0.01
CONC5	82.41 ± 11.39	1.161 ± 0.13	6.34	<0.01
CONC6	65.19 ± 11.05	0.846 ± 0.17	4.62	<0.05

* *p*-value compared with control.

**Table 5:** Product stability evaluation (mean ± SD, *n* = 3).

Code	Sampling (1st Day)		Sampling (90th Day)	
	RTP (25 ± 2 °C)		Stability Oven (40 ± 2 °C/65 ± 5%RH)	
	Diameter ± SD (nm)	Zeta Potential ± SD (mV)	Diameter ± SD (nm)	Zeta Potential ± SD (mV)
CONC1	131 ± 6.7	−28.6 ± 1.7	284 ± 11.2	−26.7 ± 1.8
CONC2	187 ± 9.1	−30.0 ± 2.1	307 ± 9.7	−29.3 ± 1.5
CONC3	163 ± 4.3	−29.4 ± 2.8	198 ± 10.5	−25.1 ± 2.8
CONC4	144 ± 3.9	−28.1 ± 3.6	173 ± 8.9	−22.7 ± 3.6
CONC5	120 ± 5.2	−27.5 ± 1.9	138 ± 4.4	−21.8 ± 2.1
CONC6	156 ± 4.5	−31.2 ± 2.4	179 ± 7.3	−28.7 ± 2.3

**Table 6:** Effect of CONC on lysosomal enzymes, C- reactive protein, rheumatoid factor, and serum cytokines in FCA-induced arthritic rats.

Treatment	AST (U/ML)	ALT (U/ML)	ALP (U/ML)	CRP (mg/lit)	RF (IU/L)	TNF-α (pg/mL)	IL-6 (pg/mL)
Normal control	33.9 ± 0.8	25.8 ± 0.9	41.3 ± 1.7	1.2 ± 0.4	-	9.7 ± 0.3	51.4 ± 0.4
Arthritic control	78.6 ± 1.5 ^#^	75.3 ± 1.8 ^#^	121.4 ± 1.8 ^#^	8.2 ± 0.6 ^#^	57.9 ± 1.6 ^#^	26.9 ± 0.6 ^#^	118.7 ± 0.6 ^#^
Voltaren gel (diclofen 1.16%)	43.1 ± 1.7 **	39.4 ± 1.3 **	56.2 ± 1.6 **	2.9 ± 0.4 **	38.1 ± 0.8 **	13.9 ± 0.4 **	58.3 ± 0.9 **
CONC5 (1.2%)	49.4 ± 1.2 **	46.9 ± 0.8 **	60.5 ± 1.4 **	6.4 ± 0.5 **	47.3 ± 1.2 **	16.5 ± 0.2 **	62.4 ± 1.1 **

Values are mean ± SEM for 6 animals. ** *p* < 0.01 vs. control group, ^#^ *p* < 0.01 when compared to normal control. Using one-way ANOVA followed by Dunnett test. Abbreviations, AST: aspartate aminotransferase; ALT: alanine transaminase; ALP: alkaline phosphatase; RF: rheumatoid factor, CRP: C- reactive protein, TNF-α: TNF: tumor necrosis factor-α; IL-6: interleukin-6.

## Data Availability

This study did not report any data.

## Sample Availability

Samples of the CO compounds are available from the authors.
